# N-Terminal Domain of Fragile Histidine Triad Exerts Potent Cytotoxic Effect in HT1080 Cells and Increases Doxorubicin Cytotoxicity

**Published:** 2019

**Authors:** Ameneh Eslamparast, Reza Abbasgholizadeh, Seyed Nasser Ostad, Mehdi Gharghabi, Mohammad Hossein Ghahremani

**Affiliations:** a *Biotechnology Research Center, Pasteur Institute of Iran, Tehran, Iran. *; b *Department of Pharmaceutical Biotechnology, Faculty of Pharmacy, Ardabil University of Medical Sciences, Ardabil, Iran. *; c *Department of Toxicology and Pharmacology, Faculty of Pharmacy, Tehran University of Medical Sciences, Tehran, Iran.*

**Keywords:** Fragile histidine triad, HT1080, Doxorubicin, Combination therapy, MTT assay, Flowcytometry

## Abstract

Fragile histidine triad (FHIT) serves a critical function as a tumor suppressor that inhibits p53 degradation by mouse double minute 2 (MDM2). The functional domains of FHIT involved in tumor inhibition was interpreted. *In-silico* screening data were employed to construct truncated forms of FHIT to assess their cytotoxic effects on the HT1080 cell line. Full FHIT expression was confirmed by western blotting and expression of two FHIT truncates were confirmed by RT-PCR. Transfection of these truncated forms into HT1080 cells showed that the N-terminal truncated form (amino acids 17-102) better inhibited proliferation than the full-length FHIT. The combined effects of these truncated forms augmented doxorubicin-induced cytotoxicity. Functional analysis demonstrated that these fragments and their combination with doxorubicin can arrest cells in the G2 phase of the cell cycle as specified by flow cytometry. The FHIT functional domains can be used as lead compounds for development of drug designs and gene transfer for cancer therapy.

## Introduction

FHIT is a putative tumor suppressor that has been eliminated or has low expression in various cancers (1). FHIT interacts with different proteins and directs the cell to apoptosis (2). It has been shown that FHIT is targeted downstream of the death receptor signaling pathway (3). The presence of FHIT increases Chk1 and Chk2 phosphorylation and cell cycle arrest following DNA damage (4, 5).

FHIT performs a key role in the regulation of MDM2 protein (6). In tumors with wild-type p53, p53 degradation is a possible mechanism that challenges apoptosis (6). Studies indicate that HT1080 expresses wild-type p53 (7, 8). It has been proposed that the interaction of FHIT with MDM2 interferes with the relationship of MDM2 and p53 to subsequently block MDM2-mediated p53 degradation (6). 

MDM2 protein directly interacts with p53 (9, 10), which is the ubiquitin ligase of p53 targets it for degradation and as abridging factor for nuclear export (11, 12). It has been shown that MDM2 protein can directly interact with FHIT (6, 13); thus, it is logical to assume a common binding site for FHIT and p53 on MDM2 or a possible interaction affecting the binding of these proteins to MDM2.

In order to find the potential interacting site, *in-silico *modeling of FHIT-MDM2-p53 was performed (14) and some of these protein domains were used to study the proliferative effect on HT1080 cells. The effect of these truncated FHIT proteins on HT-1080 proliferation and cell cycle was investigated. The truncated forms were targeted to the interaction site of FHIT-MDM2 for p53 binding (14). The effect of combining the truncated forms with doxorubicin on HT-1080 viability and cell cycle analysis was examined. This information can be used to propose novel drugs function as FHIT in the FHIT-MDM2-p53 protein interaction complex that will result in tumor repression.

## Experimental

*Cell lines and culture conditions*

The human cancer cell line HT1080 (human fibrosarcoma), human non-small cell carcinoma (NSCLC) cell line, A549 and MCF7 (human breast carcinoma cell line) were purchased from the National Cell Bank (Pasture Institute of Iran, Tehran). The cells were cultured in the RPMI1640 medium (Biosera, England) supplemented with 10% heat-inactivated fetal bovine serum (FBS; Biosera, England) and antibiotics (100 U penicillin/mL and 100 µg streptomycin/mL; Gibco, USA) and incubated at 37 °C and 5% CO_2_ in a humidified atmosphere.

*Plasmids construction (primer designing, cloning and subcloning)*

The full length FHIT/pcDNA3 construct was prepared from the FHIT/pCR2.1 plasmid which had been previously cloned and sequenced in our lab. The FHIT cDNA was directionally subcloned into the *Bam*HI/*Eco*RV site of the pcDNA3. Two truncated constructs, named FHIT^17 ^and FHIT^34^, were generated by PCR using FHIT^17^ 5´-CCCTATGTAGCGATGCTCAAAACAG

AA-3´(sense), 5´-CCTCTCACTTATCGTCATCATCCTTATAGTCTCCAGCCTTCCTG-3´ (antisense) and FHIT^34^ 5´-TGTGGCGATGGGTCATGTCCTTGTGTGC-3´ (sense), and 5´-CCTCTCACTTATCGTCATCATCCTTATAGTCTCCAGCCTTCCTG-3´ (antisense) specific primer pairs, carrying restriction enzyme sites, and then cloned into the pcDNA3 mammalian expression vector.

For proper expression, the ATG and Kozak sequence in 5’ and the stop codon in 3’ regions of the constructs were engineered, respectively. The truncated forms were amplified in a 50 µL reaction mixture containing 100 ng full length FHIT as template, 10 pmol of each primer, 0.2 mM dNTP, 1.5 mM MgCl_2_, and 0.5 U Taq polymerase in 1X PCR buffer (all from CinnaGen Co., Iran) under the following PCR conditions: initial 95 °C for 2 min and then 30 cycles at 95 °C for 30 sec, 65.8 °C (for FHIT^17^), or 69.7 °C (for FHIT^34^) for 40 sec, 72 °C for 18 sec followed by a final 10 min extension at 72 °C. The reactions were verified on a 1% agarose gel by ethidium bromide staining. 

The PCR products were purified from the gel, cloned into pGEM_Teasy (Promega, USA), and subcloned into pcDNA3 (Invitrogen, USA) in the *Eco*RI site. The generated plasmids were analyzed by agarose gel electrophoresis and DNA sequencing was performed to verify the sequences.

*Western blotting*

The cells were lysed and the proteins were extracted using a sodium dodecyl sulfate (SDS) lysis buffer. The transfected cells were lysed 24 h after transfection. The protein samples were boiled for 7 min, resolved on 12% SDS-PAGE, and electroblotted onto a polyvinylidene difluoride (PVDF) membrane (Roche, Germany) using a semidry apparatus (Peqlab, Erlangen, Germany). The blots were blocked in casein blocking buffer (1% casein in TBS and 0.05% tween 20); and incubated at 4 °C overnight, using anti-p53 (Cell Signaling Technology, USA) or anti-FHIT (Abcam, USA) as primary antibody. The primary antibody was revealed using horseradish peroxidase-conjugated secondary antibodies (1:10000; BioRad, USA) and the protein bands were detected using a chemiluminescence kit (Roche, Germany) on X-ray films (Fujifilm, Japan). 

The membranes were stripped and reprobed using β-actin antibody (1:1000; Santa Cruz Biotechnology) as the internal control. The protein bands were digitized and the band intensity was quantified using the ImageJ software (NIH, USA). 

*Transient transfection*

HT1080 cells were plated at 10^4^ cells/well in 96 wells and cultured for 24 h. At 60-70% confluence, the cells were transfected with full FHIT-pcDNA3, FHIT^17^-pcDNA3, FHIT^34^-pcDNA3, or pcDNA3 (vector control) plasmids using FuGENE6 (Roche, Germany) or Lipofectamin 2000 (Invitrogen, USA) and cultured in RPMI. The transfection efficiency was evaluated by the transfection of pEGFP-N1 in these cells.

*RT-PCR analysis*

At 24 h after transfection, mRNA expression of truncates were analyzed. Total RNA was extracted from the HT1080 cells using Tripure reagent (Roche, Germany), according to the manufacturer’s instructions. To eliminate the genomic and/or plasmid DNA contaminations, the extracted RNAs were treated with DNase I (Qiagen, Germany) for 30 min, after inactivation with 25 mM EDTA. The RNA quality was confirmed by the A260/A280 absorbance. Two micrograms of RNA were reverse-transcribed to a single stranded cDNA, using random hexamer primers and M-MLV reverse transcriptase (Fermentas, Ukraine). Expression analysis of FHIT truncated constructs was performed in a 50 µL reaction mixture containing: 2 µg cDNA, 10 pmol of each primer, 0.2 mM dNTP, 1.5 mM MgCl_2_, 0.5 U Taq polymerase, 1x PCR buffer, and 1% Q-solution (Qiagen, Germany). PCR amplification of FHIT truncates and β-actin was performed under the following conditions: initial 95 °C for 2 min and then 30 cycles at 95 °C for 30 sec, 61.4 °C (for FHIT^17^), or 60.5 °C (for FHIT^34^) for 40 sec, and 60 °C (for β-actin) for 30 sec, 72 °C for 18 sec, 15 sec, and 40 sec followed by a final 10 min extension at 72 °C. 

TheFHIT^17 ^5´- GAACTGTCCTTCGCTCTTGTG-3´ (sense), 5´-TATCGTCATCATCCTTATAGTCTCC-3´ (antisense), and FHIT^34^ 5´-ATGGGTCATGTCCTTGTGTG-3´ (sense), and 5´- TATCGTCATCATCCTTATAGTCTCC-3´ (antisense) primers were designed to amplify only the truncated constructs and to avoid amplification of endogenous FHIT. β-actin was used as the internal control.

*Drug treatment and cytotoxicity assay*

HT1080 cells were transfected (quadruplicate, n = 4) and incubated for 24 h. The cells were then treated with doxorubicin (Ebeve, Austria) at different concentrations (10, 100, and 1000 nM) for 24 h and cytotoxicity was measured by MTT assay (Sigma, UK). The change in formazan crystals was measured at OD 570 nm with OD 690 nm as a reference wavelength on the microplate reader (BioTek, USA).

*Cell cycle analysis*

HT1080 cells transfected with the FHIT constructs, the pcDNA3 empty vector, and some transfected cells were treated with doxorubicin (10 nM). The cells were then trypsinized 24 h after transfection (or 24 h after treatment), resuspended in PBS, fixed in 70% ethanol, and incubated in fluorochrome solution containing 50 mg/mL propidium iodide DNA staining buffer (Sigma, Germany). After incubation for 3 h at 4 °C, the cell cycle distribution was recorded in FL3 using flow cytometry (FACS Caliber; Becton Dickinson, USA). The distribution of 10,000 cells was determined by the Dean Jet Fox Model, using the FlowJo software (Tree Star, Inc., Standford, CA, USA). 

*Statistical analysis*

The results were analyzed using one-way ANOVA followed by the Tukey-Kramer post-hoc test. A *p*-value of less than 0.05 (*p* < 0.05) was considered significant. All the experiments were performed in triplicate and the results of the three independent experiments were reported as mean ± SE (n = 3).

## Results

*Generation of constructs*

According to the *in-silico* interaction studies on FHIT truncated forms with p53 and MDM2, the truncated forms FHIT^17^ and FHIT^34 ^were selected and constructed as described in the methods section. The full-length FHIT/pcDNA3 construct was prepared from FHIT/pCR2.1 plasmid, which had been previously cloned and sequenced in our lab.

*Transient transfection*

**Figure 1:**
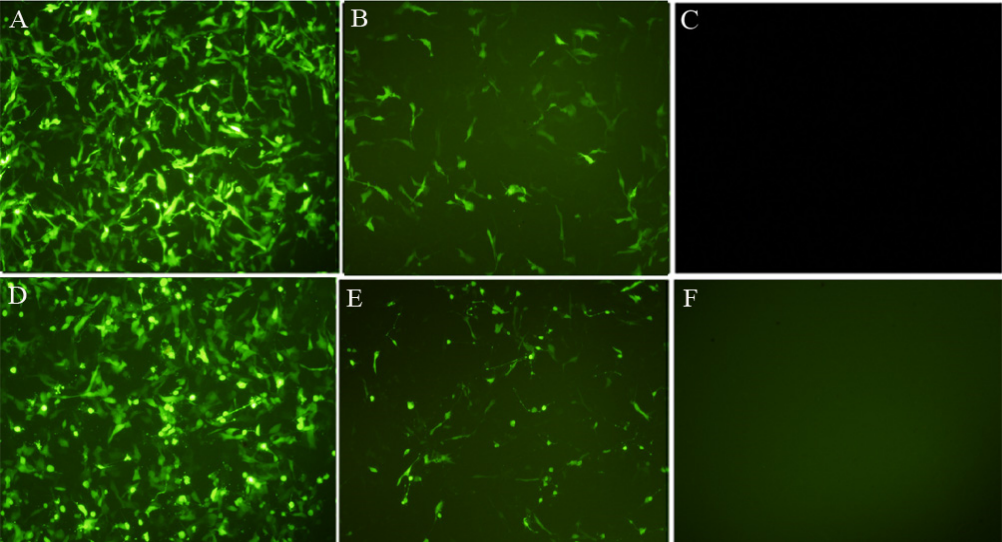
Transfection efficiency of HT1080 with EGFP. HT1080 cells were transfected with EGFP with lipofectamin 2000 (A: 24 h, D: 48 h) or FuGENE6 (B: 24 h, E: 48 h) and were compared with untransfected cells (C and F). The green fluorescent of EGFP expression was evaluated 24-48 h after transfection

Transfection efficiency was optimized by introducing pEGFP-N1 into the HT-1080 cells using Lipofectamine 2000 and FuGENE6. The transfection was analyzed using fluorescent microscopy of EGFP expression within 24 to 48 h in the transfected cells (Figure 1). The condition showing the highest expression was used for subsequent experiments.

*Expression of FHIT and p53 in HT1080 cells*

**Figure 2:**
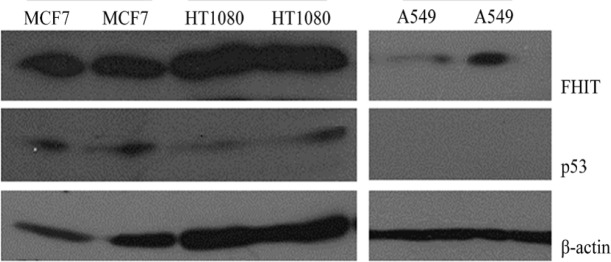
Western blot analysis of endogenous FHIT (with Abcam Anti-FHIT) and p53 expression in HT1080 and A549 cell lines. MCF7 was used as high expressing cells and β-actin was used as internal control

**Figure 3:**
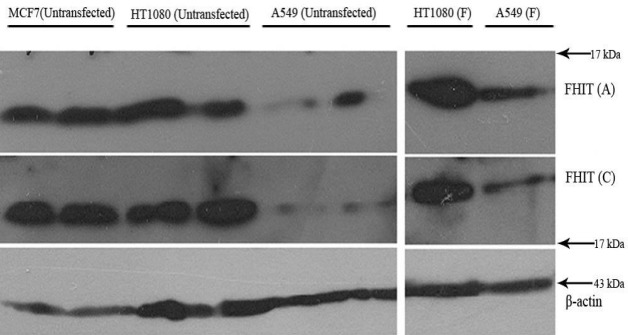
Western blot analysis of FHIT overexpression in HT1080 and A549 cell lines. Transfected (F: transfected with FHIT) and untransfected cells were lysed and subjected to western blot using anti FHIT (A = Abcam, C = Cell signaling) antibodies. MCF7 was used as high expressing cells and β-actin was used as internal control.

Western blot analysis was used to detect expression levels of FHIT and p53 in HT1080 cells. Figures 2 and 3 show that HT1080 cells endogenously expressed FHIT and p53. The A549 cells (negative control) expressed a low level of FHIT and an undetectable amount of p53. The level of FHIT increased after transfection (Figure 3F). The MCF7 cells as the positive control expressed FHIT and p53.

*RT-PCR analysis of FHIT truncated forms expression*

The mRNA level of the truncated constructs in transfected cells was evaluated to detect the expression of the truncated forms. 

**Figure 4:**
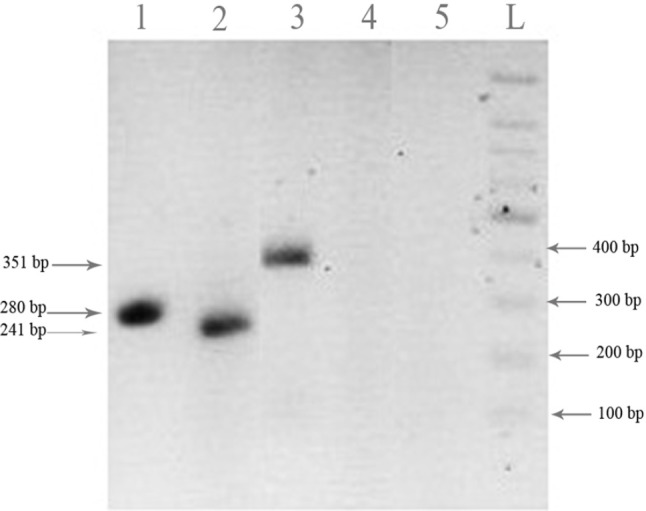
mRNA expression of FHIT truncated forms in HT1080 cell. Cells were transfected with FHIT and the truncated forms and the cells were harvested and subjected to RT-PCR analysis 24 h after transfection as described in the methods. mRNA expression of FHIT17 truncated form (lane 1), FHIT34 truncated form (lane 2) and corresponding β-actin (lane 3) were indicated. The pcDNA3 empty vector (lane 4), RT-PCR negative control (lane 5) and DNA ladder (L) were shown

The primers were designed to detect only truncated forms and not detect endogenous FHIT mRNA. Figure 4 shows that the transfected HT1080 cells expressed the two FHIT truncated forms at the mRNA level at 24 h after transfection. The fragments sizes were as expected and β-actin was used as the internal control.

*Effect of FHIT constructs on HT1080 cell viability*

The effect of FHIT and its truncated forms on HT1080 cell viability was tested. The results indicated that full-length FHIT did not change cell viability at 24 h compared to the control group (vector transfected cells). 

**Figure 5:**
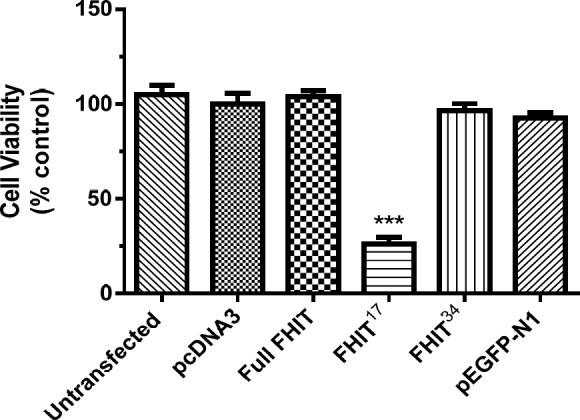
Effect of full FHIT and FHIT truncated forms constructs (FHIT17 and FHIT34) expression on HT1080 cells viability. Cells were transfected with full length FHIT, FHIT truncated forms constructs, empty vector (pcDNA3) and pEGFP-N1 (control plasmid). The cell viability was evaluated after 24 h by MTT assay. Results were calculated as percent control to pcDNA3 group and presented as mean ± SE of three independent experiments (n = 3, ***p < 0.001 compared to pcDNA3 control)

The expression of the truncated forms (FHIT^17^ and FHIT^34^) demonstrated that FHIT^17 ^inhibited viability by 50%; however, the effect of FHIT^34 ^was similar to that of full-length FHIT (*p* > 0.05; Figure 5). There was no significant difference between the cells expressing full-length FHIT and the control groups (without transfection, pcDNA3, pEGFP-N1).

*Cell viability after drug treatment and transfection*

**Figure 6:**
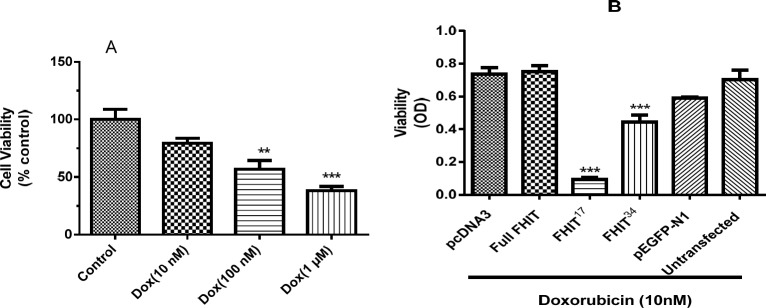
Effect of FHIT expression and doxorubicin treatment on HT1080 cell viability. (A) HT1080 untransfected cells were treated with doxorubicin for 24 h and cell viability was assessed by MTT. (B) HT1080 cells were transfected by pcDNA3 (empty vector), pEGFP-N1 (control vector), Full FHIT, and FHIT constructs (FHIT17 and FHIT34) and after 24 h, cells were treated with doxorubicin (Dox 10 nM) for 24 h. The cell viability was measured after doxorubicin treatment. The results are presented as mean ± SE of three independent experiments

FHIT expression was examined for a possible synergistic effect in combination with doxorubicin by testing the viability of HT1080 cells expressing FHIT treated with doxorubicin. It was found that doxorubicin (10 nM, 100 nM, 1000 nM) lowered HT1080 cell viability in a dose-dependent manner (Figure 6A). To test the combination, a non-effective dose of doxorubicin (10 nM) was applied to FHIT expressing cells. The results indicate that in comparison with the controls (pcDNA3, pEGFP-N1), cell viability in HT1080 cells expressing FHIT did not alter after treatment with doxorubicin (Figure 6B); however, FHIT^17^ and FHIT^34^ expressing cells showed a significant decrease in viability in the presence of doxorubicin. Interestingly, FHIT^17^ expression, which demonstrated strong growth inhibition, caused a significant change in viability in the presence of doxorubicin, and FHIT^34^ expression, which did not demonstrate strong growth inhibition, also caused a significant change in viability in the presence of doxorubicin (Figure 6B). This suggests that the truncated forms augmented the effect of doxorubicin at low doses. This is important because this combination can be expected to produce fewer side effects from the doxorubicin. These results indicated a distinct effect for the truncated forms.

*Cell cycle redistribution following expression of FHIT and its truncated forms*

**Figure 7:**
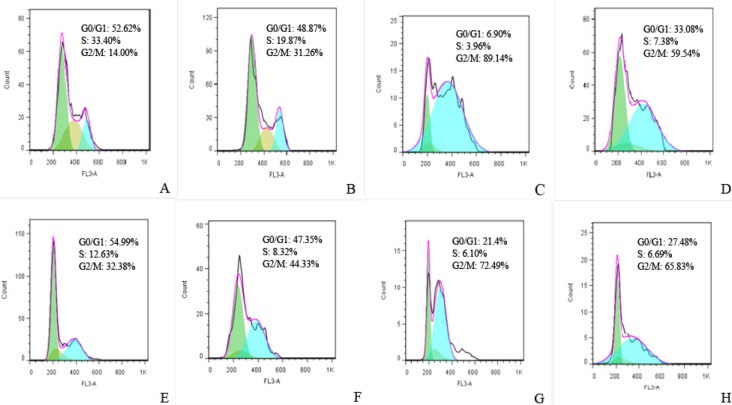
Cell cycle profiles were determined by PI staining 24 h after transfection by (A) pcDNA3, (B) full FHIT, (C) FHIT17, (D) FHIT34. Cell cycle profiles were determined by PI staining 48 h after transfection by (E) pcDNA3, (F) full FHIT, (G) FHIT17, (H) FHIT34 and 24 h after treatment with the same concentration of doxorubicin in viability test. The results of three independent experiments are shown (G0/G1 = green, S = brown, G2 = turquois)

Flow cytometry revealed that both FHIT truncates induced significant G2/M arrest. FHIT^17^ expression produced a stronger accumulation of cells in the G_2_/M phase and decreased the cell population in the G1 and S phases relative to vector control (Figure 7). Interestingly, FHIT did not perform as strongly as the truncated forms. FHIT also arrested cells in the G2/M phase at 24 h.

## Discussion

The present *in-silico* results indicate that FHIT can interact with MDM2 and compete with p53 for interaction with MDM2, inducing cell death (14, 15). The docking findings reveal that structures containing β6-7 and α1 motifs maintained low total energies for MDM2/FHIT interaction and p53/FHIT interaction (14, 15). The role of FHIT^17^ and FHIT^34 ^truncated proteins in viability of HT1080 cell was investigated. It was found that FHIT^17^ showed a strong cytotoxic effect for HT1080 cells, suggesting a critical role of this truncated FHIT. Full-length FHIT showed no significant cytotoxic effect; more importantly, this protein inhibited proliferation in HT1080 cells that express high levels of FHIT. This effect was evidently domain-dependent, since FHIT^34^ showed no significant effect. 

*In-silico* experiments showed that FHIT^17 ^interacted with MDM2 and optimized MDM2 fraction with less total energy than the full FHIT model (14, 15). Besides, all structures that can change the MDM2/p53 interaction site in shape or electrostatically can inhibit MDM2/p53 interaction and p53 degradation with MDM2. MDM2 protein directly interacts with p53 (9, 10) and controls p53 function by binding to its transcription domain, adding ubiquitin to contribute to its degradation and binding to p53 to assist its nuclear export (11, 12). Studies have demonstrated the interaction of p53 and FHIT (6, 13) and their possible association (16). Former research has also confirmed that the significant fraction in p53-MDM2 interaction is amino acids 18-26 and the significant fraction of MDM2 is amino acids 23-119 (17). Because the FHIT^17 ^truncated form had a strong cytotoxic effect in the presence of highly endogenous FHIT, a better interaction with MDM2 and a strong inhibitory effect on p53 association with MDM2 can be concluded. 

The possibility of a synergistic effect for FHIT truncated forms in combination with doxorubicin was examined. FHIT^17^ and FHIT^34^ expressing cells showed significantly reduced viability in the presence of doxorubicin. The results pointed to an interesting finding that FHIT^34^, which showed no cytotoxicity when expressed in HT1080 cell, was potentiated cytotoxicity in combination with doxorubicin (Figure 4B). This effect is of great importance, since non-effective dose of doxorubicin was used in these cells. By increasing doxorubicin dose to 100 nM, higher cytotoxicity in combination with doxorubicin was observed; however, the effect was not significant. It has been reported that FHIT expression potentiates doxorubicin cytotoxicity in the bladder carcinoma tissue (18) and gastric cancer cell line (19). However, in non-small cell lung carcinoma (H460) lacking FHIT, FHIT expression reduces doxorubicin and etoposide-induced cytotoxicity (20). These results suggest a strong effect of FHIT truncated form with low doses which can lower side effects during the treatment. 

Based on our results, the FHIT truncated forms increased the cytotoxicity of doxorubicin at low doses. This effect can be explained by combination of different mechanisms of cytotoxicity induced by FHIT and doxorubicin. The doxorubicin induces cytotoxicity by intercalating with DNA (21, 22) and inhibition of Topoisomerase II (23). On the other hand, FHIT seems to induce cell death via inhibition of p53 degradation (6), inhibition of Akt pathway (24), induction f p38 kinase (5), and activation of Caspase 9 (25). Thus, the combine mechanism seems to induce cell death in low doses.

Studies show that FHIT and doxorubicin individually can arrest most cells in the G2/M phase. Functional analysis showed that FHIT fragments and their combination with doxorubicin arrested HT1080 cells in the G2 phase of the cell cycle as confirmed by flow cytometry. These results indicate that FHIT^17^ can inhibit proliferation and arrest the G2 phase and that FHIT^17^and FHIT^34^ can strongly potentiate the effect of doxorubicin and strongly arrest the G2 phase of cell cycle. These reports suggest a critical role for FHIT for DNA damage-induced cell death. The present findings demonstrate a distinct effect for FHIT truncated protein induction of cytotoxicity. The results also show that FHIT^17^ inhibited proliferation and that FHIT^17 ^and FHIT^34^ strongly potentiated the effect of doxorubicin. These results are important for the design of therapeutic strategies for cancer treatment.
